# Capacitor based topology of cross-square-switched T-type multi-level inverter

**DOI:** 10.1038/s41598-024-53568-1

**Published:** 2024-02-07

**Authors:** Ali Seifi, Seyed Hossein Hosseini, Mehrdad Tarafdar Hagh, Majid Hosseinpour

**Affiliations:** 1 Faculty of Electrical and Computer Engineering, University of Tabriz, Tabriz, 51666-16471 Iran; 2 Engineering Faculty, Near East University, 99138 Nicosia, Turkey; 3 Department of Electrical and Computer Engineering, University of Mohaghegh Ardabili, Ardabil, 56199-11367 Iran

**Keywords:** Electrical and electronic engineering, Solar energy

## Abstract

In this paper, a new topology is introduced for capacitor-based multi-level inverters. The proposed topology is based on combination of two Cross-Square-Switched T-Type inverters. This structure can be generalized in two modular and cascaded modes. In the cascaded mode, higher voltage levels are produced with low power switches. The main features of the proposed topology include the level generation without the utilization of the H-bridge module, the low number of switching components, a lower number of DC voltage sources, and low total blocking voltage. Besides, in the proposed topology, the number of conducting switches in the current path for each different voltage level is low, which leads to a conduction loss decrement. The loss simulations are performed, and the results are presented. A study provides a detailed comparison of the proposed topology in terms of various parameters. In this paper, the nearest level modulation switching, which is low-frequency switching, is utilized to generate voltage levels. To confirm the performance of the proposed topology, a simulation was performed with MATLAB/Simulink software, and a laboratory sample was implemented. Comparative results, simulation results, and implementation results indicate the appropriate performance of the proposed structure in different steady-state and dynamic conditions.

## Introduction

Since interconnecting a two-level inverter to a high-voltage network is not possible, with the growing development of renewable energy resources such as photovoltaic arrays and wind energies, the demand for the progress of high-voltage, high-power inverters have increased. Multi-level inverters (MLIs) are a suitable solution for this purpose, where new topologies are being developed. MLIs include power electronics switches and DC sources that generate different voltage levels from a combination of voltage sources. The main feature of MLIs compared to two-level inverters is generating voltage waveforms with better quality and close to the sine waves, which this improvement in voltage waveform will reduce the total harmonic distortion. Other features of MLIs are switching loss reduction, low voltage stress of switches, high-quality output power, less electromagnetic interference, etc.^1–5^.

Traditional MLIs mainly consist of three categories, which include neutral point clamped (NPC), flying capacitor (FC), and cascading H bridge (CHB). NPC and FC MLIs utilize multiple capacitors to generate voltage levels, making these configurations challenging in regulating the voltage of these capacitors. Moreover, with increasing the number of voltage levels at the output of these MLIs, the number of capacitors and power switches increases^6–9^. Due to the increment in the number of components, the power circuit of these MLIs will be complicated, and it will also involve a complex control scheme. CHB MLIs consist of some H-bridge modules, which is connected in series. These MLIs do not require clamped or flying capacitors. Additionally, CHB MLIs have advantages such as modularity, simple control, reliability, and utilizing low-power switches^10,11^. The CHB MLIs are divided into symmetric and asymmetric topologies in terms of equal or unequal input voltage sources. In the first category, the DC voltage sources have the same values, whereas this configuration has good modularity. Nevertheless, in the second category, the DC voltage source values are different and unequal. In this type of configuration, the number of output voltage levels rises using the number of switches equal to the symmetric topology. Traditional MLIs have one major drawback, and that is a large number of switches, which becomes a significant issue at higher voltage levels^11,12^.

In MLIs, the quality of the output voltage improves with an increasing number of output voltage levels^10^. The principal challenge for MLIs is the number of switches, the number of gate-drivers, and the number of circuit components, which severely increase with the increasing output voltage levels. This increment in the number of components will increase the volume, cost, and complexity. Accordingly, it is tried to minimize the number of circuit components of MLIs for high voltage levels^13–20^.

The necessity for multiple DC voltage sources is also a significant challenge for MLIs. This challenge is significant in CHBs, which have many voltage sources. In some studies, capacitors have been utilized instead of some DC voltage sources to reduce the number of DC voltage sources in the CHB structure^13–15^. In these configurations, capacitor voltage control is complex, and the process of charging and discharging the capacitor voltage may not be complete, and the output voltage may include unwanted harmonics. Some MLIs use only one DC voltage source to generate multi-level voltage, known as a switched-capacitor structure. In some of these configurations, the procedure of charging capacitors is complex and does not have a modular structure. In these configurations, a DC voltage source provides all the power required, which may not be suitable for high power applications. In addition, in these configurations, because the output power must be supplied by a DC source, the DC input current is high, which increases the conduction losses of the switches and thus reduces the efficiency of these configurations^10^.

In MLIs used for high power, utilizing a structure based on a single DC source is not appropriate, and to provide high power, using some DC voltage sources is inevitable. In some topologies, a DC-DC interface circuit is utilized to increase the number of DC voltage sources^9^. The DC-DC interface circuit can increase one voltage source to several voltage sources. However, this circuit has circuit elements such as inductors, capacitors, diodes, and switches that increase the volume of the circuit. So, this approach is not a practical solution to supply the required number of DC sources. Furthermore, the total efficiency of the converter will be reduced simply because of adding such a single-input multiple-output DC-DC converter.

The proposed multi-level inverter offers an improved arrangement in which the switches are designed to maximize the number of output voltage levels with limited circuit components. This paper introduces a cross-square-switched T-type (CSST-type) topology that is capable of operating with both equal and unequal sources. The proposed topology in unequal configuration can be implemented in both incremental and decremental combinations, which further increases the output voltage levels. The number of conducting switches at each of the voltage levels is small in the proposed topology, which will reduce the conduction losses. Additionally, the voltage stress of the proposed topology switches is low, and high output power can be achieved with low power switches. The proposed topology reduces the number of DC voltage sources to 2 by providing the voltage division between the capacitors and requires only 2 DC voltage sources. Moreover, the proposed topology can be developed in series and provide a cascaded structure in which the number of output voltage levels will be increased significantly.

“CSST-type topology” section of this paper discusses the principles of CSL-type MLI operation, including basic cell configuration, generalized structure configuration, voltage sources reduction, cascaded structure, and symmetric and asymmetric topologies. Power losses and efficiencies are calculated in “Loss-thermal analysis” section. Comparative studies are presented in “Comparing the proposed MLI configuration with other configurations” section. simulation results followed by laboratory results are presented in Sect. “Simulation and laboratory results”.

## CSST-type topology

### The proposed structure

**Figure 1:**
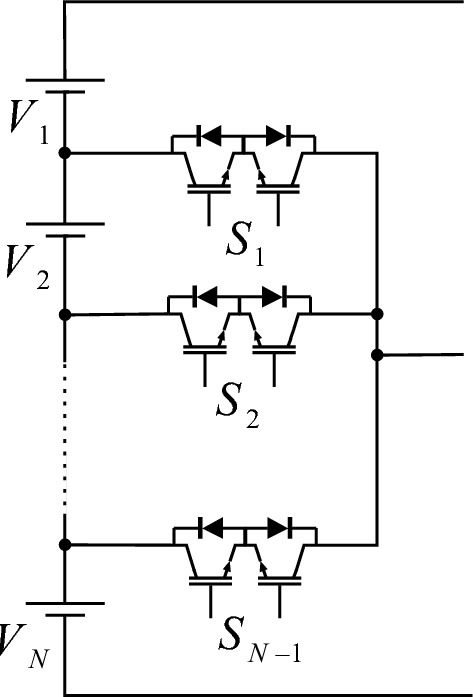
T-type module.

In The CSST configuration utilizes the T-type module, which is displayed in Fig. 1. As Fig. 1 demonstrates, the T-type module consists of N DC sources, and N − 1 bidirectional switches. The t-type module only produces positive voltage levels and requires an H bridge to generate negative voltage levels.

**Figure 2:**
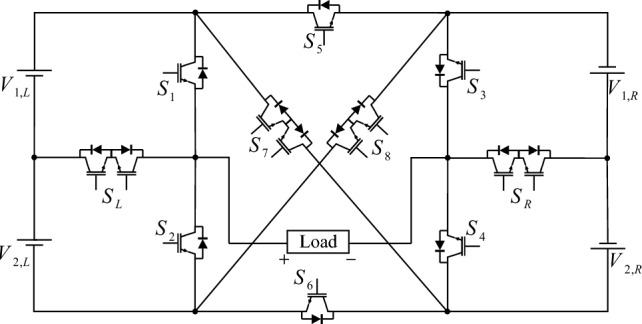
The proposed CSST-type structure configuration.

The proposed CSST-type structure configuration consists of two back-to-back three-level T-type modules. T-type modules are connected with a cross-square-switched module. The configuration of the proposed CSST-type structure is shown in Fig. 2. The proposed CSST-type structure consists of two parts: the right part, which is labeled R, and the left part, which is labeled L. Due to the inverse connection of the T-Type module on the left and right, the output voltages of these two modules are added together at the output of the proposed structure, and the number of output voltage levels increases. The proposed CSST-type structure can be used in both equal or unequal modes. In the unequal mode, the sources can be incremental/decremental combinations, thus providing more output levels. As the number of output voltage levels increases, the voltage THD decreases considerably.

**Figure 3:**
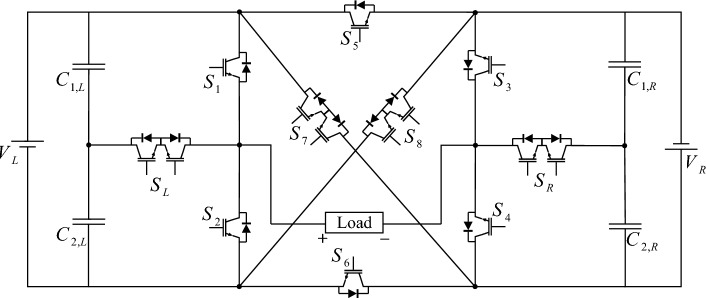
The proposed CSST-type structure configuration with reduced resources.

In the proposed CSST-type structure, each T-type module utilizes two DC sources. In this case, the number of resources is significant, and providing this number of resources is a big challenge. To reduce the number of sources in the proposed CSST-type cell configuration, voltage division is used between capacitors. With this approach, the number of structure resources is declined to 2. Figure 3 presents the proposed CSST-type cell configuration by reducing the number of sources by voltage division method.

In the DC source reduction configuration, the capacitor is used to divide the voltage of the DC sources, and the voltage of the capacitors is equal to:

1 $$V_{{C_{1,L} }} = V_{{C_{2,L} }} = \frac{{V_{L} }}{2}$$

2 $$V_{{C_{1,R} }} = V_{{C_{2,R} }} = \frac{{V_{R} }}{2}$$

The proposed CSST-type structure does not require a side circuit (one-input multi-output DC-DC converter) to balance the voltage of the capacitors by reducing the number of DC sources and using capacitors. In other words, the proposed structure can automatically adjust the voltage of the capacitors. The proposed CSST-type structure consists of 2 sources that can be designed in equal and unequal modes. In equal topology, the proposed structure can produce nine voltage levels. In the equal topology, the proposed CSST-type structure can generate voltage levels only as an incremental combination of capacitor voltages. In other words, in generating different output voltage levels, it is only possible to add the voltage of the capacitors together. In this topology, the V_R_ and V_L_ voltage sources are equal to V_DC_, and different modes of generating voltage levels are presented in Table 1.

**Table 1:** Different switching modes of CSST-type structure.

Level	Switching state (1 = ON, 0 = OFF)									
	*S*_*1*_	*S*_*2*_	*S*_*3*_	*S*_*4*_	*S*_*5*_	*S*_*6*_	*S*_*7*_	*S*_*8*_	*S*_*R*_	*S*_*L*_
+ 4	1	0	1	0	0	1	0	0	0	0
+ 3	0	0	1	0	0	1	0	0	0	1
	1	0	0	0	0	1	0	0	1	0
+ 2	0	0	0	0	0	1	0	0	1	1
	0	1	1	0	0	1	0	0	0	0
	1	0	0	1	0	1	0	0	0	0
+ 1	0	1	0	0	0	1	0	0	1	0
	0	0	1	0	0	0	0	1	0	1
	1	0	0	0	0	0	1	0	1	0
	0	0	0	1	0	1	0	0	0	1
0	1	0	0	1	0	0	1	0	0	0
	1	0	1	0	1	0	0	0	0	0
	0	1	0	1	0	1	0	0	0	0
	0	1	1	0	0	0	0	1	0	0
-1	0	0	1	0	1	0	0	0	0	1
	0	1	0	0	0	0	0	1	1	0
	0	0	0	1	0	0	1	0	0	1
	1	0	0	0	1	0	0	0	1	0
-2	0	1	1	0	1	0	0	0	0	0
	1	0	0	1	1	0	0	0	0	0
	0	0	0	0	1	0	0	0	1	1
-3	0	1	0	0	1	0	0	0	1	0
	0	0	0	1	1	0	0	0	0	1
-4	0	1	0	1	1	0	0	0	0	0

As Table 1 displays, the (*S*_1_, *S*_2_), (*S*_3_, *S*_4_), and (*S*_5_, *S*_6_) switch pairs act as complementary and never turn on together. Besides, the number of switching modes of the CSST-type structure in generating 0, ± 1, ± 2, ± 3 voltage levels has more than one switching, which is a helpful feature in space vector modulation. In space vector modulation, the switching state is selected to have the most minor change in switching states to reduce switching losses^21^.

The total blocking voltage (TBV) of the Multi-level structure is calculated from the maximum blocking voltage (MBV) of the switches. The maximum voltage across the switch in the off mode determines the voltage stress of the switches. The maximum blocking voltage of the proposed CSST-type switches is given by the following equations:

3 $$V_{S1} = V_{S2} = V_{L}$$

4 $$V_{S3} = V_{S4} = V_{R}$$

5 $$V_{SL} = V_{L}$$

6 $$V_{SR} = V_{R}$$

7 $$V_{S5} = V_{S6} = V_{L} + V_{R}$$

8 $$V_{S7} = V_{S8} = V_{L} + V_{R}$$

In the equal topology, since the size of the *V*_*R*_ and *V*_*L*_ voltage sources is equal to *V*_*DC*_, the proposed *TBV* of the proposed CSST-type cell is obtained as follows:

9 $$TBV_{EQ} = V_{S1} + V_{S2} + V_{S3} + V_{S4} + V_{S5} + V_{S6} + V_{S7} + V_{S8} + V_{SL} + V_{SR} = 14V_{DC}$$

**Figure 4:**
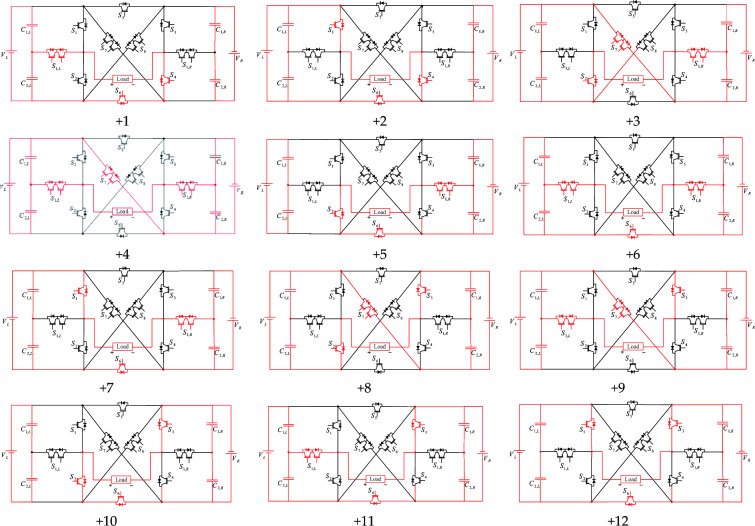
Positive output voltage levels of the proposed CSST-type structure.

In the unequal topology, the ratio of the size of the left and right voltage sources of the proposed CSST-type structure is 1:5. In other words, the *V*_*L*_ voltage source is equal to *V*_*DC*_, and *V*_*R*_ is equal to 5*V*_*L*_ = 5*V*_*DC*_. In an unequal topology, the combination of voltage sources is incremental/decremental. In other words, in the different output voltage level generation, in addition to the possibility of adding the voltage of the capacitors together, it is also possible to subtract the voltage of the capacitors from each other. This leads to a significant increase in the number of output voltage levels, and the unequal topology of the proposed structure can produce 25 levels. Figure 4 displays the different modes of positive voltage level generation. This figure illustrates the current path and light switches in red. The negative voltage levels are obtained in the same way.

The maximum blocking voltage of the proposed CSST-type structure switches is obtained by Eq. (3)–(8), which for an unequal topology, the *TBV* value is equal to:

10 $$TBV_{UEQ} = V_{S1} + V_{S2} + V_{S3} + V_{S4} + V_{S5} + V_{S6} + V_{S7} + V_{S8} + V_{SL} + V_{SR} = 42V_{DC}$$

The value of *TBV* in both equal and unequal modes is low due to the number of voltage levels produced, indicating that the voltage range of the switches is low.

### The proposed modular structure

**Figure 5:**
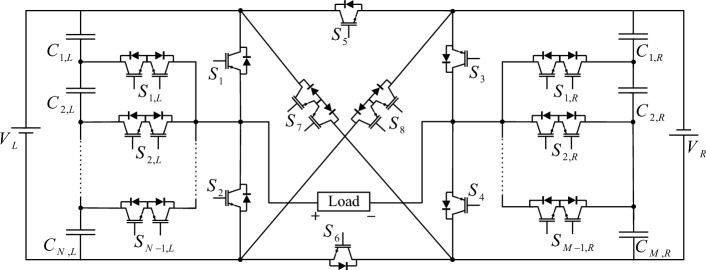
The proposed modular CSST-type structure.

The proposed CSST-type structure can be generalized in both modular and cascading methods. The configuration of the modular CSST-type structure is presented in Fig. 5. In this configuration, T-type cells are generalized modularly, for which bidirectional switches are added to T-type cells.

The modular CSST-type configuration has several distinctive features that are mentioned. (1) To extend the output levels to higher levels, only one bidirectional switch is added to the structure. Each bidirectional switch requires only one driver. Thus, the number of drivers in this configuration will be low at high voltage levels. (2) The number of DC voltage sources in this configuration is only two. (3) This configuration can be designed with both equal and unequal sources. (4) The number of active switches and current conductors at different voltage levels is only three switches, which will reduce the conduction losses.

The number of switches (*N*_*S*_), number of gate drivers (*N*_*GD*_), number of capacitors (*N*_*C*_), and number of sources (*N*_*DC*_) of the modular CSST-type configuration are as follows:

11 $$N_{S} = 2(N + M) + 10$$

12 $$N_{D} = (N + M) + 8$$

13 $$N_{C} = N + M + 2$$

14 $$N_{DC} = 2$$

*N* and *M* are the numbers of bidirectional switches on the left and right of the modular CSST-type configuration. In the equal topology, the voltage source ratio of the modular CSST-type configuration is 1:1. In the unequal topology of the modular structure, the number of source voltages can be selected based on the following:

15 $$V_{L} = V_{DC}$$

16 $$V_{R} = (2N + 3)V_{L} = (2N + 3)V_{DC}$$

**Figure 6:**
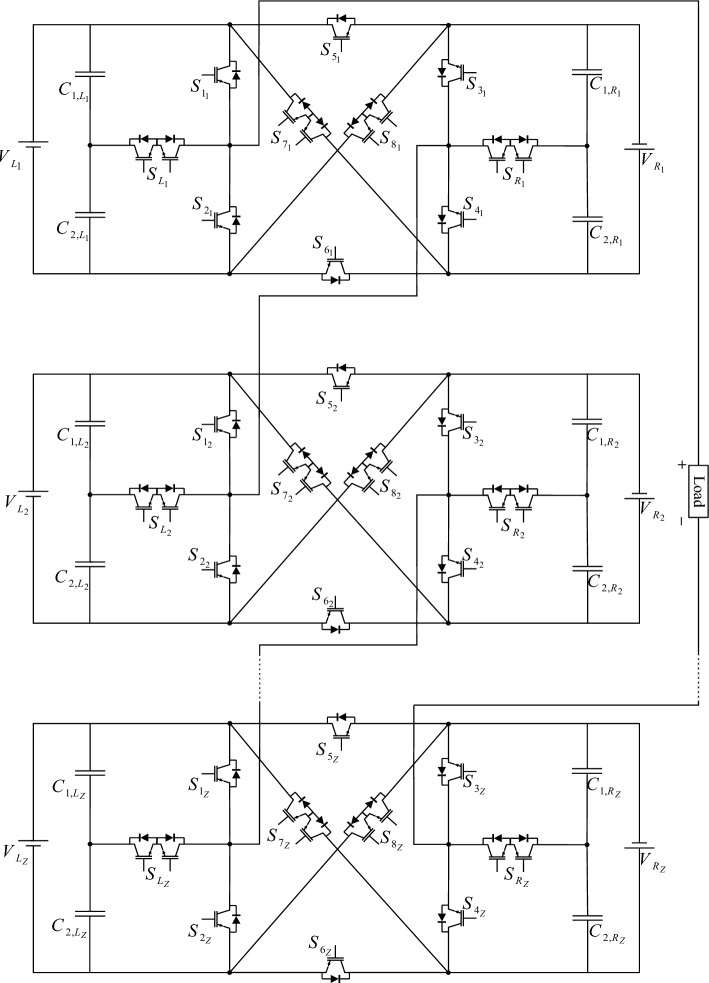
CSST-type cascade configuration.

The CSST-type cascade configuration consists of the *Z* number of the basic CSST-type structure shown in Fig. 6. The purpose of providing cascade configuration is to achieve high voltage levels using low voltage and power switches.

The number of switches (*N*_*S*_), number of gate drivers (*N*_*GD*_), number of capacitors (*N*_*C*_), and number of sources (*N*_*DC*_) in the cascading CSST-type configuration are as follows:

17 $$N_{S} = 14Z$$

18 $$N_{D} = 10Z$$

19 $$N_{C} = 4Z$$

20 $$N_{DC} = 2Z$$

To determine the size of voltage sources of cells, many algorithms can be used. Table 2 presents some examples of possible algorithms. In this table, $\hat{V}_{{O_{z} }}$ is the output voltage level peak of the *Z*th cell. In the third algorithm, the number of levels increases sharply. With two CSST-type cells, it is possible to generate 625 voltage levels at the output.

**Table 2:** CSST-type cascade configuration cells voltage source size determination algorithms.

Proposed algorithm	The magnitude of DC voltage sources	*N*_*L*_
1st algorithm	$$\begin{gathered} V_{{L_{1} }} = V_{{L_{2} }} = ... = V_{{L_{Z} }} = V_{DC} \hfill \\ V_{{R_{1} }} = V_{{R_{2} }} = ... = V_{{R_{Z} }} = V_{DC} \hfill \\ \end{gathered}$$	$8Z + 1$
2nd algorithm	$$\begin{gathered} \,V_{{L_{1} }} = V_{{L_{2} }} = ... = V_{{L_{Z} }} = V_{DC} \hfill \\ V_{{R_{1} }} = V_{{R_{2} }} = ... = V_{{R_{Z} }} = 5V_{DC} \hfill \\ \end{gathered}$$	$24Z + 1$
3rd algorithm	$$\begin{gathered} V_{{L_{1} }} = V_{DC} ,\,V_{{R_{1} }} = 5V_{DC} \hfill \\ V_{{L_{1} }} = 25V_{DC} ,\,V_{{R_{1} }} = 125V_{DC} \hfill \\ V_{{L_{Z} }} = (2(\hat{V}_{{O_{1} }} + \hat{V}_{{O_{2} }} + ... + \hat{V}_{{O_{Z - 1} }} ) + 1)V_{DC} ,\,V_{{R_{1} }} = 5V_{{L_{Z} }} \hfill \\ \end{gathered}$$	$25^{Z}$

## Loss-thermal analysis

The power semiconductor devices, such as DC-DC converters, rectifiers, matrix converters, and two-level or multi-level inverters, generally have two types of loss, which are: conduction loss (*P*_*c*_) and switching loss (*P*_*sw*_). The conduction loss is due to the internal resistance and voltage drop in the ON state of the semiconductor devices. This loss consists of the conduction loss of IGBT or MOSFET, and its anti-parallel diode, denoted by (*P*_*c,s*_) and (*P*_*c,d*_), respectively. These losses are calculated by the following Equations:

21 $$P_{C,S} = \left[ {V_{S} + R_{S} i^{\beta } (t)} \right]i(t)$$

22 $$P_{C,D} = \left[ {V_{D} + R_{D} i(t)} \right]i(t)$$

where *V*_*s,ON*_ and *V*_*d,ON*_ are the voltage drop when the switch or its anti-parallel diode is turned on. The resistances *R*_*s*_, *R*_*d*_, are the internal resistance of the switch or its anti-parallel diode, and α is a constant coefficient that depends on the specifications of the switch. These parameters are prepared in the datasheet of the switches by the manufacturer. The following Equation is used to calculate the average conduction loss of all switches and their anti-parallel diodes in an output period:

23 $$\overline{{P_{c} }} = \sum\limits_{j = 1}^{{N_{s} }} {\frac{1}{2\pi }} \int\limits_{0}^{2\pi } {\left[ {V_{s,ON} i(t) + R_{s} i^{\alpha } (t)} \right]d(t)} \, + \sum\limits_{j = 1}^{{N_{d} }} {\frac{1}{2\pi }} \int\limits_{0}^{2\pi } {\left[ {V_{d,ON} i(t) + R_{s} i^{2} (t)} \right]d(t)}$$

Another part of the semiconductor power loss is switching loss. The switching loss is due to the non-ideal performance of power semiconductor devices. In order to calculate the switching loss, it is assumed that the voltage and current of the switch change linearly when it is turned on and off. Therefore:

24 $$P_{s} = \left[ {\sum\limits_{x = 1}^{{N_{s} }} {\left( {t_{s,ON} E_{s,ON} + t_{s,OFF} E_{s,OFF} } \right)} } \right]f_{s}$$

where *t*_*s,ON*_ and *t*_*s,OFF*_ are the time intervals required to turn a switch on and off, *E*_*s,ON*_ and *E*_*s,OFF*_ are the energy dissipation of the switch at the moments of turning on and turning off, and *f*_*s*_ represents the switching frequency. So, the total loss of a switch (*P*_*T*_) is the sum of the conduction loss of the switches and their anti-parallel diodes, as well as the switching loss, presented in Eq. (25).

25 $$P_{T} = P_{C,T} + P_{S,T}$$

Also, the efficiency of the converter is calculated according to (25):

26 $$\eta = \frac{{P_{Output} }}{{P_{Output} + P_{T} }}$$

**Figure 7:**
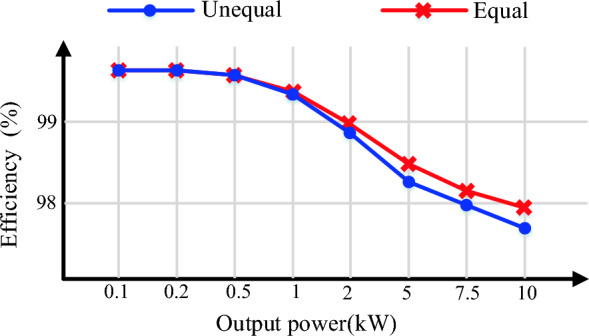
The efficiency of the proposed CSST-type structure in terms of output power.

**Figure 8:**
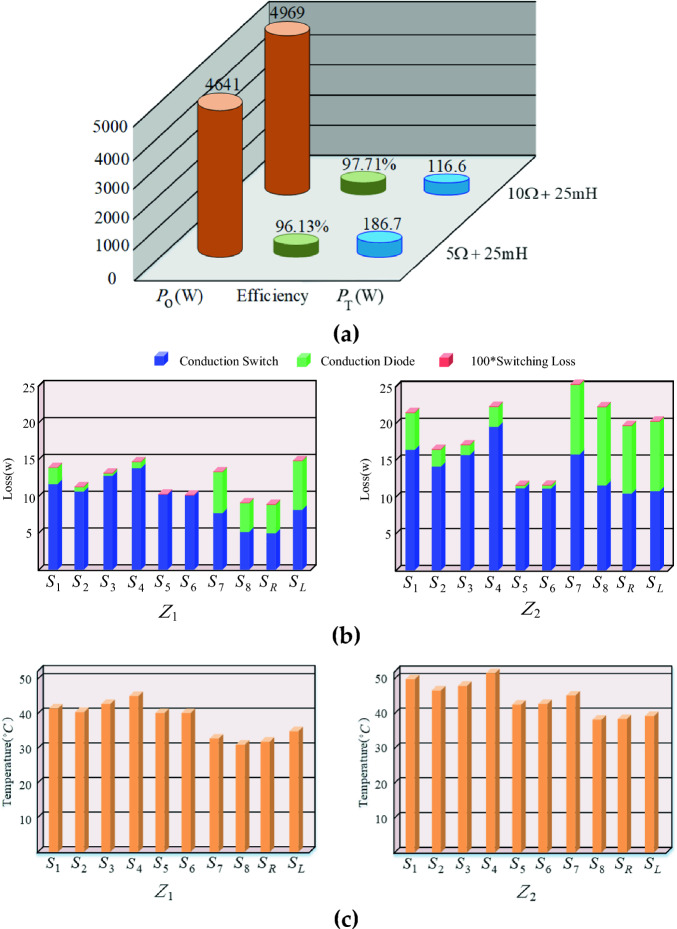
(**a**) Output power, tola loss, and efficiency of proposed MLI, (**b**) Conduction and switching loss of the power electronics components, (**c**) Temperature of the switches for the ambient temperature of 25 °C.

The switching and conduction loss considering the thermal model of power electronics components is simulated in MATLAB/Simulink software. The performance of the proposed CSST-type structure is investigated in both symmetric and asymmetric topologies at pure resistance load, where the peak voltage of the load is considered to be 400 V. The parameters of the IGBT IKFW60N60DH3E switch is used for this simulation. The simulation is conducted based on Pulse Width Modulation (PWM) switching pattern. The efficiency of the proposed CSST-type structure for both symmetric and asymmetric topologies in terms of output power from light load to full load is shown in Fig. 7. The total loss, the output power, and the efficiency of the proposed CSST-type structure in symmetric topology for two different output loads (Z_1_ = 10 Ω + 25mH), (Z_2_ = 5 Ω + 25 mH) are demonstrated in Fig. 8a. In addition, the loss and temperature of the switches are demonstrated separately in Fig. 8b,c.

## Comparing the proposed MLI configuration with other configurations

In this section, a comparison is made to demonstrate the advantages of the proposed CSST-type configuration. The comparison is based on the number of components in the proposed configuration compared to the classic multi-level inverter topologies and new reduced components. In this section, the output voltage levels of inverters (*N*_*L*_) are compared to the number of switches (*N*_*Switch*_), number of gate drivers (*N*_*GD*_), number of DC sources (*N*_*DC*_), number of capacitors (*N*_*C*_), and *TBV* of the switches. Table 3 compares the proposed CSST-type structure with other new structures. In this table, in addition to the mentioned parameters, the number of active switches (*N*_*AS*_), the number of diodes (*N*_*D*_), the demand for an H-bridge to generate negative polarity, the need for a DC-DC interface circuit to reduce the number of DC sources, number of switches to the number of output levels (N_Switch_/N_L_), and DC voltage ratio (V_DC_ ratio) are also presented.

**Table 3:** Comparison of the proposed CSST-type structure with recently reduced component topologies.

	Mode	*N*_*L*_	*N*_*Switch*_	*N*_*GD*_	*N*_*DC*_	*N*_*C*_	*TBV*(*V_*dc*_)	*N*_*AS*_	*N*_*D*_	*N*_*Switch*_*/N*_*L*_	*H-bridge*	*V*_*DC*_ ratio	*DC-DC*
CHB	EQ	9	16	16	4	0	16	8	0	1.78	Yes	1:1	No
	UEQ	27	12	12	3	0	52	6	0	0.45	Yes	1:3	No
^14^	UEQ	17	10	10	2	6	38	5	6	0.59	No	1:3	Yes
^15^	EQ	11	11	10	2	4	34	4	2	1	No	1:1	Yes
	UEQ	19	11	10	2	4	50	4	2	0.58	No	1:2	Yes
^16^	EQ	11	12	10	4	0	22	4	0	1.09	No	1:1	No
	UEQ	17	12	10	4	0	40	4	0	0.7	No	1:3	No
^17^	EQ	7	10	9	3	0	14	4	0	1.43	No	1:1	No
	UEQ	15	10	9	3	0	34	4	0	0.67	No	1:3	No
^18^	EQ	11	12	10	2	6	22	4	3	1.09	No	1:1	Yes
	UEQ	17	12	10	2	6	40	4	3	0.7	No	1:3	Yes
^19^	UEQ	31	14	10	6	0	72	5	0	0.45	No	1:4	No
^20^	EQ	7	12	9	3	0	18	4	0	1.71	No	1:1	No
	UEQ	11	12	9	3	0	26	4	0	1.09	No	1:2	No
^21^	UEQ	25	18	12	7	0	78	3	0	0.72	No	1;2	No
^22^	EQ	9	10	8	4	0	18	3	0	1.11	No	1:1	No
	UEQ	17	10	8	4	0	36	3	0	0.59	No	1:3	No
^23^	EQ	7	8	8	3	0	12	3	0	1.14	No	1:1	No
	UEQ	11	8	8	3	0	26	3	0	0.73	No	1:2	No
^24^	UEQ	11	8	7	3	0	22	3	0	0.73	No	1:2	No
^25^	UEQ	13	10	8	4	0	32	3	0	0.77	No	1:2	No
^26^	EQ	7	10	10	3	0	20	5	0	1.43	Yes	1:1	No
	UEQ	11	10	10	3	0	34	5	0	0.9	Yes	1:2	No
^27^	UEQ	17	12	9	4	0	40	3	0	0.7	No	1:3	No
^28^	EQ	13	12	11	1	4	26	6	4	0.92	No	1:1	No
^29^	EQ	9	10	10	2	2	10	4	8	1.11	No	1:1	No
	UEQ	25	10	10	2	2	60	4	8	0.4	No	1:5	No
^30^	UEQ	49	12	12	2	2	128	6	0	0.24	No	1:7	No
^31^	UEQ	17	12	12	2	4	40	4	0	0.7	No	1:3	No
Proposed	EQ	9	14	10	2	4	14	3	0	1.55	No	1:1	No
	UEQ	25	14	10	2	4	42	3	0	0.56	No	1:5	No

**Figure 9:**
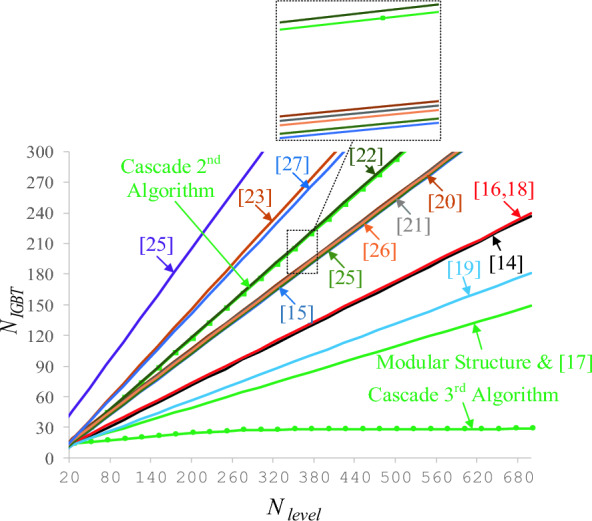
Comparison of the number of switches in the proposed CSST-type structure with other structures.

Switches are a critical element in the structure of multi-level inverters, which increase the output voltage levels, and the number of switches. As the number of switches and circuits of multi-level inverters grows, the cost, complexity, and size of the circuit increases. Figure 9 compares the number of proposed CSST-type structure switches with the number of output levels in the unequal mode. Figure 9 demonstrates that the CSST-type topology has a smaller number of switches than other similar structures, decreasing the cost and complexity of the circuit.

**Figure 10:**
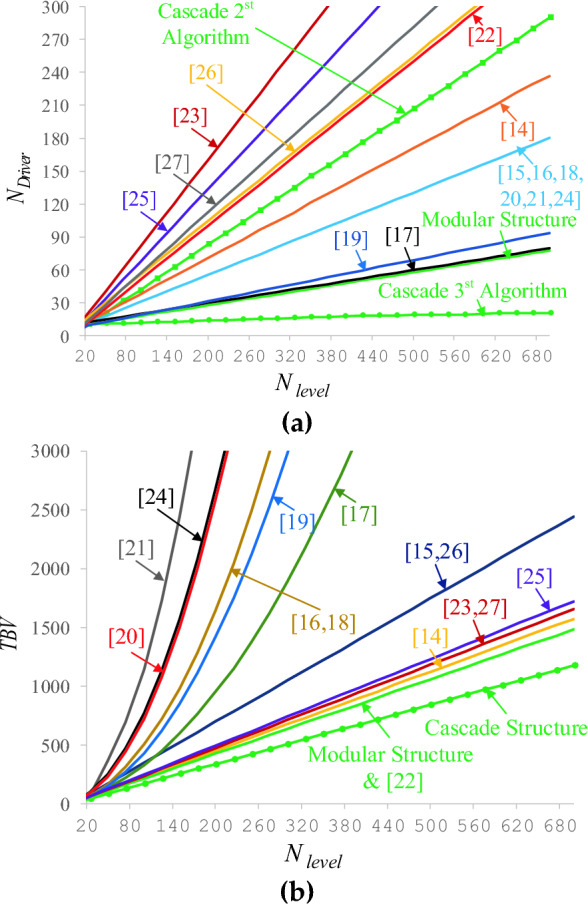
(**a**) Comparison of the number of gate-driver, (**b**) comparing TBV.

A large number of drivers in multi-level inverters increases the cost. Each switch requires a gate driver to be turned on and turned off, which boosting the gate pulses of the micro-controller is the gate driver's duty. Bidirectional switches will need only one driver if designed as a common-emitter. In a modular CSST-type configuration, a bidirectional switch is required to generate two additional voltage levels. Therefore, the number of drivers in the proposed CSST-type configuration is low. Figure 10a compares the number of proposed CSST-type structure drivers with other topologies. The number of drives used in the proposed CSST-type structure is lower than other topologies. TBV of the proposed CSST-type structure is compared with other topologies in Fig. 10b. It can be seen from Fig. 10b that the TBV value of the proposed CSST-type modular structure is lower than other structures.

**Figure 11:**
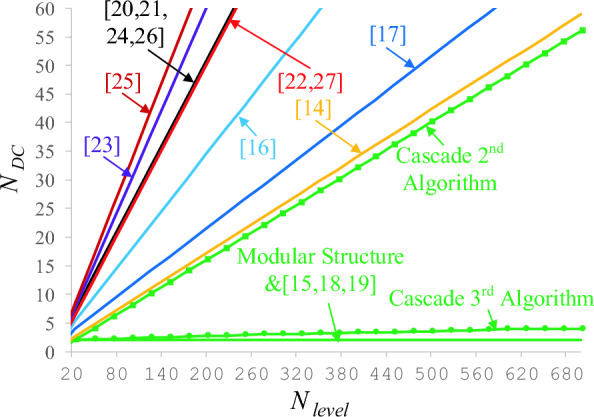
Comparing the number of DC voltage sources of the proposed CSST-type structure and similar ones.

The number of independent voltage sources in multi-level inverters is one of the significant challenges. Providing a large number of sources in multi-level inverters is a huge problem. Figure 11 displays the number of independent voltage sources of the proposed CSST-type structure with other topologies. Based on this figure, the number of independent voltage sources of the proposed topology is very low. The number of sources of the proposed structures in^15,18,19^ is equal to the proposed topology. However, in these structures, the interface circuit is used to balance the voltage of the capacitors. The interface circuit consists of circuit elements such as switches, diodes, inductors, and capacitors, which increase the complexity of the whole system and decrease its efficiency. Besides, the number of switches, drivers, and TBV of the structures presented in^15,18,19^ are high.

## Simulation and laboratory results

**Figure 12:**
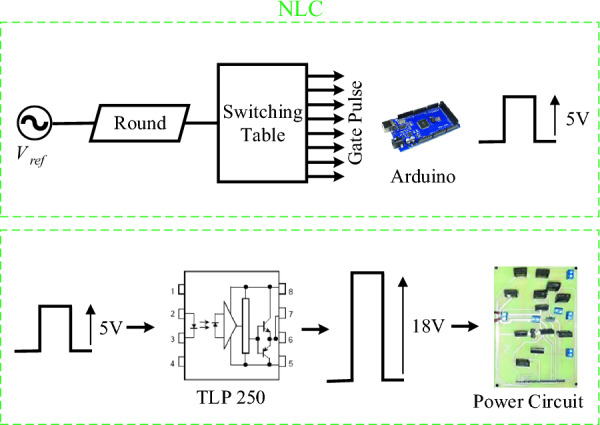
Control block in a laboratory sample.

**Figure 13:**
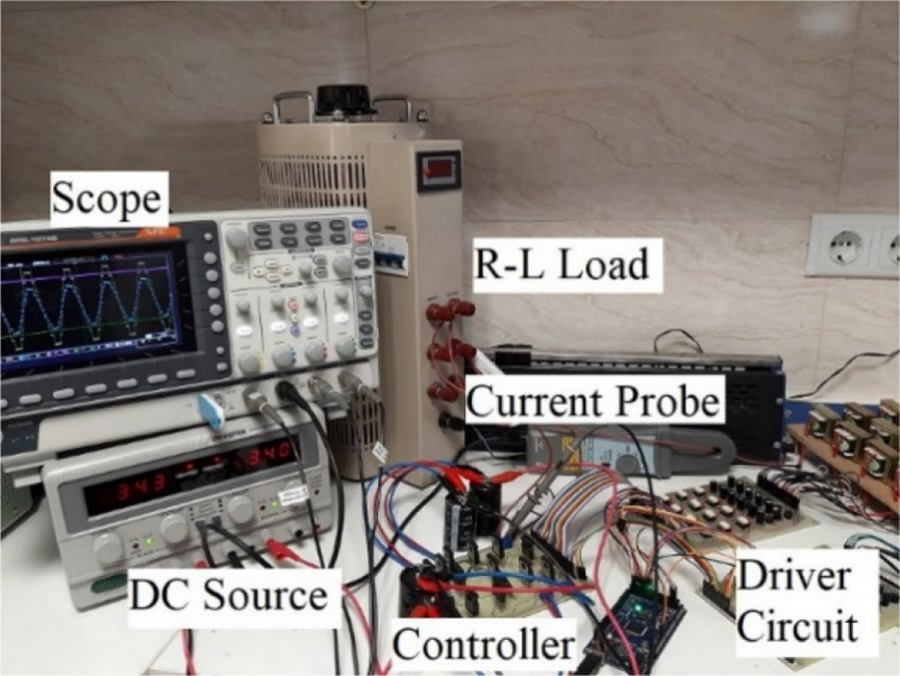
Prototype of the proposed CSST-type structure.

In this section, simulation and laboratory results are presented to confirm and validate the proposed CSST-type structure. The simulated and implemented version of the proposed CSST-type structure is controlled and switched by the nearest surface modulation (NLM) method. The results of the proposed CSST-type structure are presented for both equal and unequal modes, and information on circuit parameters is presented in Table 4. Figure 12 displays the control method in the laboratory sample. As this figure shows, the range of pulses generated by the Arduino microcontroller is 5 V, and to drive the MOSFETs, a pulse signal with an approximate range of 15 V is required, which is done by the driver circuit with the help of TLP250 Optocoupler. Figure 13 presents a laboratory sample of the proposed CSST-type structure.

**Table 4:** Circuit parameters of the proposed CSST-type cell.

	Equal	Unequal
*V*_*in*_	*V*_*L*_=*V*_*R*_=36* V*	*V*_*L*_=12* V*, *V*_*R*_=60* V*
$\hat{V}_{o}$	72 V	72 V
*N*_*L*_	9	25
*f*	50 Hz	
Controller	Arduino Mega 2560	
Optocoupler	TLP 250	
Switch	MOSFET IRFP 460	
C	1000 µm

**Figure 14:**
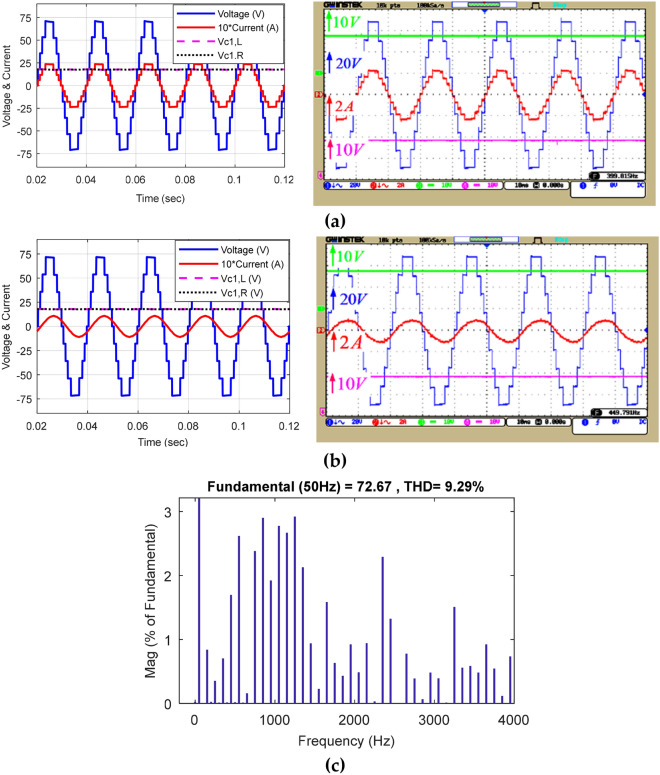
Voltage and current waveform of the load and voltage of the capacitors in equal mode: (**a**) pure resistive output load, (**b**) resistive-inductive output load, (**c**) load voltage THD.

**Figure 15:**
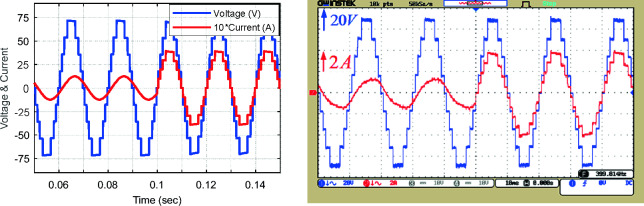
Load voltage and current waveform in the equal mode under dynamic conditions of output load change.

Figure 14 demonstrates the voltage and current waveforms of the capacitors in the equal mode and the harmonic voltage spectrum. Figure 14a displays the simulation and implementation results of the proposed CSST-type structure at a purely resistive load (Z = 30 Ω); Fig. 14b displays similar results in the resistive-inductive output load (Z = 60 Ω + 100mH), and Fig. 14c demonstrates the harmonic voltage spectrum of the load. The nine-level voltage THD of the load in the equal mode is 9.29%, and all harmonic degrees are less than 3%. Figure 15 displays the voltage and current waveform of a load in the equal mode under dynamic conditions and exchange for an instantaneous change in the size of the output resistive-inductive impedance (Z = 53 Ω + 80 mH) to a pure resistive load (Z = 18 Ω). According to this figure, the proposed structure can feed the variable output load correctly in the dynamic conditions of instantaneous change of output load size.

**Figure 16:**
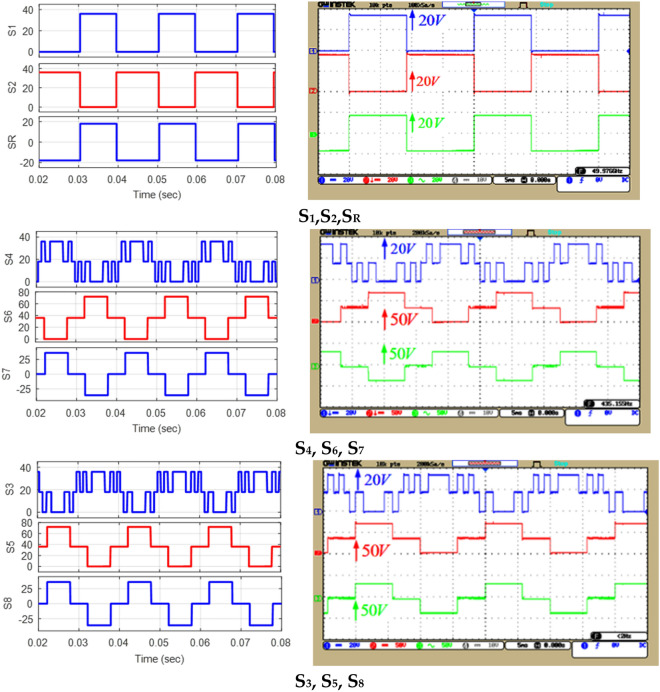
The reverse voltage of the switches of the proposed CSST-type structure.

Figure 16 displays the reverse voltage of the switches of the proposed CSST-type structure. Based on these figures, the maximum blocking voltage of each switch and the total blocking voltage of the converter can be evaluated. Based on this figure, the correctness of Eqs. (3)–(8) are available.

**Figure 17:**
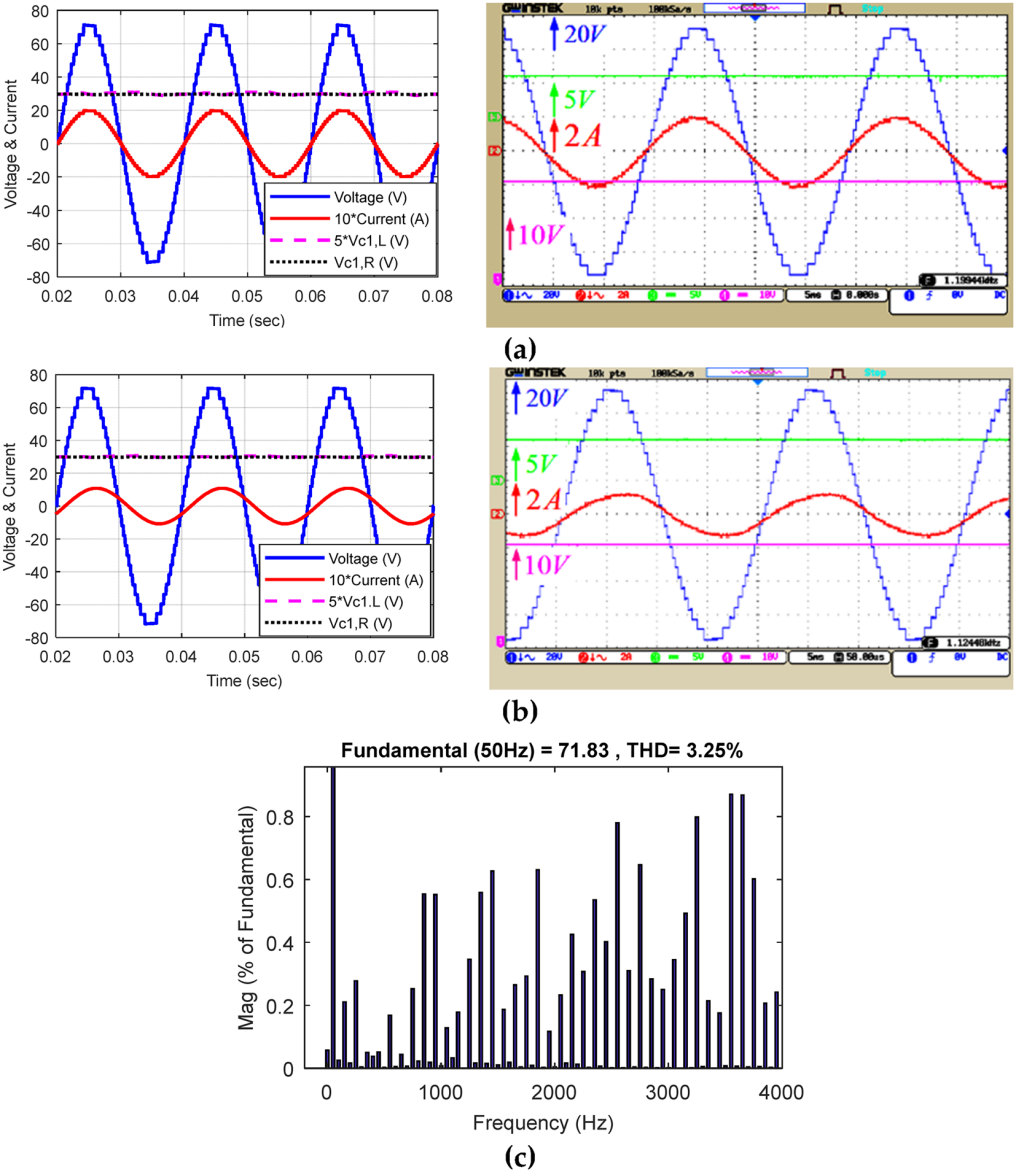
Voltage and current waveform of the load and voltage of capacitors in unequal mode: (**a**) pure resistive impedance, (**b**) resistive-inductive impedance, (**c**) load voltage THD.

Figure 17 displays the voltage and current waveform of the load and the voltage of the capacitors, and the harmonic voltage spectrum for unequal topology according to the values in Table 4. Figure 17a presents the simulation and implementation results of the proposed CSST-type structure in pure resistive load with Z = 36 Ω, Fig. 17b shows the same results in resistive-inductive load with Z = 60 Ω + 90 mH, and Fig. 17c displays the harmonic voltage spectrum for unequal topology. In this case, the number of load voltage levels with the same number of circuit components has increased to 25 levels, and the output voltage THD has decreased by 3.25%. The THD value of the load voltage in unequal topology can meet the IEEE std. 519–2014 standard. According to this standard, the maximum allowable distortion of a certain harmonic in a low-voltage network (*V* ≤ 1 kV) is 5%, and in a medium voltage network (1 kV ≤ *V* ≤ 69 kV) is 3%. Moreover, the maximum allowable total harmonic distortion in a low-voltage network can be equal to 8%, and in a medium-voltage network can be equal to 5%^32^. In such cases, the volume and frequency of the output filter are significantly reduced and can lead to a reduction in the cost of the output filter of the multi-level inverter.

**Figure 18:**
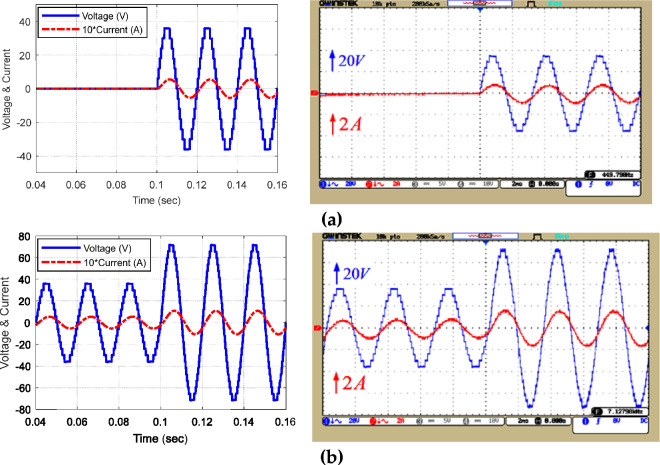
Voltage and current waveform of the load in the unequal mode for an instantaneous change of modulation index (**a**) from 0 to 0.5 and (**b**) from 0.5 to 1.

Figure 18 displays the voltage and current waveform of unequal topology for dynamic conditions in exchange for instantaneous change of the modulation index. Based on this figure, the proposed structure executes the dynamic conditions of modulation index change well and produces the appropriate output voltage in these dynamic conditions.

**Figure 19:**
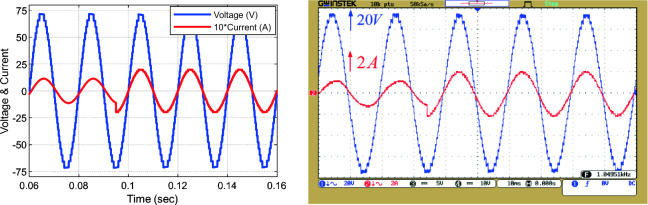
Load voltage and current waveform in the unequal mode under dynamic change of output load.

Figure 19 shows the output voltage and current waveform in the unequal mode under dynamic load change conditions (Z = 53 Ω + 80 mH to Z = 18 Ω). According to the dynamic test results (change in output load and modulation index) validate the real-time operation of the proposed MLI.

Comparing simulation results and laboratory results reveal that the resulting waveforms are well matched. The proposed structure correctly generates the required multi-level voltage under dynamic conditions such as changing the size, type of output load, and the modulation index. The voltage of the capacitors of the structure is well balanced without the necessity for a side circuit.

## Conclusion

In this paper, a new capacitor-based multi-level inverter (MLI) topology is introduced to reduce components, including the number of switches and independent voltage sources. The proposed topology is derived from a combination of two Cross-Square-Switched T-Type (CSST-type). The proposed inverter topology can be utilized for both equal and unequal sources. This topology can also be generalized in two modular and cascading modes, in which higher voltage levels can be achieved using switches with a low voltage/power range in cascading mode. The advantages of the proposed topology include positive and negative level generation without the H bridge, the low number of switching devices, the lower number of DC sources, and the acceptable blocking voltage. Besides, in the proposed structure, there are a small number of current-conducting switches at different voltage levels, which increases the efficiency of the converter. The efficiency of the proposed topology at the output power of 10 kW is about 98%, and the loss-temperature analysis of each switch indicates the uniform temperature distribution of each of the switches of the proposed structure. Additionally, in the unequal THD mode, the output voltage is only 3.25% which can pass the IEEE standard. To confirm the performance of the proposed topology, simulation and laboratory results are presented in different load modes, dynamic load change, and modulation index change modes, and the correct performance of the proposed structure is illustrated.

## Data Availability

All data generated and analysed during the current study are available from the corresponding author on reasonable request.
